# Melancholia

**Published:** 1908-07-25

**Authors:** 


### Mental Diseases.

### MELANCHOLIA.

The feelings of the melancholiac can be, to a certain extent, appreciated by everyone who has had a bad attack of " liver " with constipation, and has noticed the accompanying mental phenomena, usually termed " the blues." The feeling that one is good for nothing in this world, but merely a nuisance to others; the feeling of being looked at by every passer-by; the morbid introspection and self-condemnation for trivial mistakes in the past which come to appear enormities, can all be seen reproduced on a large scale in the melancholiac. The distinguishing feature of melancholia, namely, mental pain, though it may not be very prominent, is always present in these attacks, and forms, so to speak, the all-pervading atmosphere of the state of consciousness. Anyone who has experienced these sensations knows, too, that the proper cure—calomel and saline—will, in the course of a few hours, bring about a change to normal cheerfulness, and a complete disappearance of all those morbid feelings which disordered bodily functions have caused to become lurid.

The true attack of melancholia is of gradual onset, and is often well advanced before the extent of the trouble is recognised. The patient becomes more and more absorbed and introspective until he is so self-obsessed that he can give attention to nothing except his imaginary troubles. Delusions are common and of a painful character, those of a religious or hypochondriacal type being specially frequent; and these delusions give rise to refusal of food, and homicidal and suicidal tendencies. Of these symptoms, refusal of food occurs in a large number of cases and may be obstinate, whilst homicidal and suicidal tendencies have to be specially watched for in every case, and are often only revealed by cunningly planned attempts. Fear of impending evil is at times an obsession, and is likely to cause much restlessness. Sleeplessness is commonly found, and often the little rest obtained is broken by unpleasant and distressing dreams. Sensibility to physical pain appears to be deadened, sometimes to such an extent that any bodily suffering is welcomed as a relief to mental pain. Of physical symptoms, constipation appears very early and lasts through the attack, usually accompanied by digestive troubles and loss of body weight. The pulse is slow and full, and blood pressure is raised. Many cases show excessive sebaceous secretion, especially seen on the face, which is at times so extreme that the patient appears to have been oiled.

The prognosis in simple acute melancholia is good, although even cases which are apparently mild at times run a long course. Subsequent attacks are unfortunately fairly common, and the prognosis in each subsequent attack becomes more unfavourable. Cases due to toxic causes and organic brain lesions, or accompanied by chronic physical disease, are of bad prognosis.

The first indications for treatment are to clear the bowels, to guard against further constipation, and to see that sufficient nourishment is taken. Forcible feeding has to be resorted to frequently in melancholia, and, if necessary, four feeds a day must be given. In the early stages rest in bed may be tried, but moderate exercise in the open air should shortly be used. Attempts to " cheer up " the patient will usually cause mental suffering; he should rather be got to do some manual work which will not tire the body, but will occupy his mind and keep it off his own thoughts. Constant supervision is essential on the part of those looking after the case on account of the almost invariable, though possibly unrevealed, tendency to suicide or homicide; a tendency which those who have not had special training with the insane are but too apt to ignore. This tendency must be specially borne in mind when the advisability of travelling is discussed. Cool baths or douches are sometimes useful, and also massage of the limbs. Electricity by the interrupted current must be cautiously used, as much suffering may be caused owing to the diminished reaction to physical pain. An acute physical illness will often clear up the mental symptoms, even in old standing cases which are considered incurable.

As regards drugs, veronal gr. v. and paraldehyde ʒj. to ʒij. are the best hypnotics. Opium and morphia are useful, lessening the mental pain and as a rule regulating the bowels rather than causing constipation. They do not lead to habit in these cases, and even where large doses are being given, are stopped without difficulty. The best preparation to employ is liquor opii sedativus, ♏15 being given twice a day to start and increased until ʒj. or ʒij. a day are being given. Spirit of sulphuric ether is usually given in 15♏ doses with the opium or morphia. Thyroid gland may be given to cause physical reaction, and is often found to act well. During its early administration the patient should be kept in bed, and the reaction of the heart and temperature noted. If small doses are found of no avail, it may be increased with care. During convalescence tonic treatment is indicated.

